# Supplementary material for: Mental Health First Aid guidelines for helping a suicidal person: a Delphi consensus study in the Philippines
Source: Int J Ment Health Syst. 2010 Dec 20;4:32. doi: 10.1186/1752-4458-4-32 (PMC3017011; doi:10.1186/1752-4458-4-32)
Supplement: Additional file 1 — Table of data showing the items included in the Delphi survey and the endorsement levels from the Filipino panel members. [file 1752-4458-4-32-S1.DOC]

| **PHILIPPINES[[1]](#footnote-2)** |  |
| --- | --- |
| Section 1: Warning signs  Accepted items: | *% Agreement to insert item in guidelines* |
| Threatening to hurt or kill themselves | 97.1 |
| Looking for a way to kill themselves (e.g. seeking access to pills or poisons, weapons or other means), including asking information about possible suicide methods (e.g. “would 100 mg of this kill me?) | 100.0 |
| Talking or writing about death, dying or suicide (including making unexpected jokes about these topics) | 91.2 |
| Expressions of hopelessness | 93.1 |
| Describing themselves as a burden to others or expressing feelings of guilt or shame (e.g., stating that others will be better off without them) | 93.1 |
| Acting recklessly or engaging in risky activities, seemingly without thinking | 85.7 |
| Feeling trapped, like there is no way out | 86.2 |
| Withdrawing from friends, family or society | 86.2 |
| Sudden or dramatic increase in depressed mood | 91.1 |
| Giving away valued possessions and getting affairs in order including asking others to take on responsibility for the care of people or pets | 93.1 |
| Expressing a lack of reasons for living, or having no purpose in life | 91.1 |
| *An important warning sign for suicide is a person expresses in words or actions that they feel worthless or that their life is worthless. | 96.6 |
| *An important warning sign for suicide is a person expresses in words or actions the desire or hope that they will die (including praying that God may take their life). | 93.1 |
| *An important warning sign for suicide is if a person is engaging in self-injurious behaviour such as cutting, poisoning or hitting their head against the wall. | 100.0 |
| *An important warning sign for suicide is if a person stops life-saving medical treatments/medications. | 86.2 |
| *An important warning sign for suicide is if a person expresses in words or actions that suicide is the only solution to their problems. | 96.5 |
| *An important warning sign for suicide is if a person expresses in words or actions loss of interest of the things they used to be interested in. | 92.8 |
| *An important warning sign for suicide is if a person contacts people (e.g. family members and/or people they have not spoken to in a long time) to say goodbye, make amends or ask for forgiveness. | 96.5 |
| **Rejected items:** |  |
| No longer talking or writing about death, dying or suicide (including no longer making jokes about these topics) | 38.3 |
| Expressions of rage, anger, seeking revenge | 41.2 |
| Starting or increasing cigarette, alcohol or drug use | 41.1 |
| Unusually high levels of anxiety or agitation | 61.8 |
| Sleep disturbance – inability to sleep, or needing to sleep all the time | 55.9 |
| Dramatic change in behaviour, mood, appearance (positive or negative) | 61.7 |
| Sudden recovery from depressed mood | 47.0 |
| A lack of interest in or plans for the future | 61.8 |
| Significant change in the level of religious interest or preoccupation with afterlife | 61.8 |
| *An important warning sign for suicide is if a person is not doing usual important tasks, such as household chores, going to work or school. | 62.1 |
| *An important warning sign for suicide is if a person expresses in words or actions a sense of shame (e.g. from failure or loss). | 62.1 |
| *An important warning sign for suicide is if a person increases the levels of interpersonal conflict (e.g. arguing with spouse or family members). | 31.0 |
| *An important warning sign for suicide is if a person is refusing to eat. | 58.6 |
| *An important warning sign for suicide is a person becomes violent and/or hostile. | 37.9 |
| *An important warning sign for suicide is if a person stares blankly into space (“tulala”). | 34.5 |
| *An important warning sign for suicide is if a person expresses in words or actions that no one fully understands what they are going through. | 55.2 |
| *An important warning sign for suicide is if a person expresses in words or actions fear of God’s punishment if they killed themselves. | 48.3 |
| *An important warning sign for suicide is if a person is saying they wish or intend to see or speak to someone who is dead (e.g., a deceased family member). | 69.0 |
| *An important warning sign for suicide is if a person is making somatic complaints which cannot be explained by a medical problem. | 20.7 |
| *An important warning sign for suicide is if a person is expressing in words or actions that they have lost their religious faith. | 51.7 |
|  |  |
| Section 2: Identification of suicide risk  Accepted items: |  |
| The first aider should be able to recognise the warning signs of suicide. | 100.0 |
| The first aider should tell the suicidal person that they care and want to help | 100.0 |
| If the first aider thinks someone might be having suicidal thoughts, they should ask that person directly if they are considering suicide. | 91.2 |
| The first aider should not avoid using the word ‘suicide’. It is important to discuss the issue directly, without expressing fear or negative judgement. | 97.0 |
| If the first aider clearly states that thoughts of suicide may be associated with a treatable disorder, this may instil a sense of hope for the suicidal person. | 88.2 |
| The first aider should understand that the threat of suicide may indicate that a person is trying to communicate to the first aider how badly he or she feels. | 100.0 |
| If the first aider appears confident in the face of a suicide crisis, it may have a reassuring effect for the suicidal person. | 100.0 |
| The first aider should allow the suicidal person to discuss their feelings. | 100.0 |
| If the person is using drugs or alcohol, the first aider may not be able to believe them if they say they are not suicidal. | 89.3 |
| *The first aider should try to determine whether there is anything important in the person’s life which may reduce the immediate risk of suicide (e.g. attachments to children). | 100.0 |
| **Rejected items:** |  |
| The first aider should be aware that if a person is not suicidal, asking them might put the idea of suicide in their head. | 35.3 |
| The first aider should be aware that if a distressed person says they are not suicidal, they probably are not. | 44.1 |
| If the person is psychotic, the first aider may not be able to believe them if they say they are not suicidal. | 64.7 |
| *The first aider should be aware that the absence of a belief in a God may increase the risk of suicide. | 65.5 |
|  |  |
| Section 3: Assessing seriousness of the suicide risk **Accepted items:** |  |
| The first aider should establish whether the person has definite plans and intentions to take his life as opposed to vague suicidal notions such as “what’s the point,” or “I can’t be bothered going on”. | 97.1 |
| The first aider should ask the suicidal person if they have a plan for suicide. | 91.1 |
| The first aider should ask the suicidal person what method they intend to use. | 94.1 |
| The first aider should find out if the suicidal person has already taken steps to acquire the means to end their life. | 97.0 |
| The first aider should ask the suicidal person if they have decided when they will carry out their plan. | 94.1 |
| The first aider should ask the suicidal person if they have been using drugs or alcohol. | 100.0 |
| The first aider should ask the suicidal person if they have ever made a suicide attempt in the past. | 100.0 |
| The first aider should ask the suicidal person if they have ever known anyone who has died by suicide. | 88.3 |
| The first aider should take all thoughts of suicide seriously. | 94.1 |
| The first aider should be aware that the lack of a plan for suicide is not sufficient to ensure safety. | 94.2 |
| The first aider should avoid asking the suicidal person if they have a ‘mental illness’, instead asking if they are receiving help for any emotional or mental health problems. | 100.0 |
| *The first aider should ask significant others (e.g. family members or religious leader) whether the person has made a previous suicidal attempt. | 100.0 |
| *The first aider should ask the suicidal person if there are people they can turn to when they need help or support. | 100.0 |
| **Rejected items:** |  |
| The first aider should ask the suicidal person if they are really serious or just looking for attention. | 53.0 |
| The first aider should ask the suicidal person if they are suffering from any mental illness. | 64.7 |
| The first aider should avoid discussion of any mental health problems experienced by the suicidal person, focussing instead on the reasons behind the suicide crisis. | 67.7 |
| *The first aider should ask the suicidal person if they have experienced a change in their spiritual/religious practices or beliefs (e.g. an increase or decrease in prayer or church attendance). | 75.0 |
|  |  |
| **Section 4: Initial assistance**  **Accepted items:** |  |
| If it is known that the person is suicidal, the first aider must call a doctor, psychiatrist or other mental health professional right away. | 91.2 |
| The first aider must call the mental health emergency team. | 88.3 |
| The first aider should contact whoever is responsible for responding to psychiatric emergencies. | 91.2 |
| If the suicidal person is psychotic, the first aider should call the police or mental health team immediately. | 88.2 |
| If the suicidal person is using drugs or alcohol, the first aider should call the police or mental health team immediately. | 88.3 |
| If the suicidal person is consuming drugs or alcohol, the first aider should discourage them from consuming any more. | 86.2 |
| The first aider must not leave someone who is feeling suicidal on their own. | 85.3 |
| The first aider does not have to be with the suicidal person all the time, but should check on them regularly. | 88.2 |
| If the suicidal person has a weapon, the first aider should try to take it away from them. | 93.1 |
| If there is any risk to the first aider, for example, if the suicidal person has a gun or other weapon or is agitated, the first aider should not attempt to intervene, and should call emergency services. | 91.2 |
| The first aider should work collaboratively with a suicidal person to ensure their safety, rather than acting alone to prevent suicide at any cost. | 85.3 |
| The first aider must keep in mind that they may not be successful in preventing suicide. | 85.3 |
| The first aider should try to engage other people from the suicidal person’s social network in suicide prevention. | 100.0 |
| *The first aider should know the phone numbers of suicide hotlines, emergency services and mental health professionals. | 100.0 |
| *The first aider should take the suicidal person in the nearest safe place (e.g. church, hospital, or police station). | 89.7 |
| *The first aider should take the person to a doctor, psychiatrist or other mental health professional as soon as possible. | 93.1 |
| *The first aider should ask the suicidal person if they would like the first aider to contact someone for them, such as a friend, family member or trusted religious leader. | 96.5 |
| *The first aider should help the suicidal person understand that they have control over their suicidal thoughts. | 89.6 |
| *The first aider should remain calm and in control when communicating with a suicidal person. | 100.0 |
| *When talking to the suicidal person, the first aider should use the person’s belief systems and values to encourage them to change their mind about suicide. | 89.7 |
| *The first aider should consider the suicidal person’s spiritual/religious beliefs and refer to these to try to prevent the person from taking their life. | 82.1 |
| *The first aider should listen non-judgmentally to the suicidal person. | 100.0 |
| **Rejected items:** |  |
| The first aider should contact the person’s spiritual or religious leader, if they have got one. | 58.6 |
| The first aider should contact a traditional or alternative (i.e. non-Western) healer. | 20.6 |
| If the suicidal person is consuming drugs or alcohol, the first aider should try to take it away from them. | 64.7 |
| If the suicidal person has a weapon, the first aider should not try to take it away from them. | 14.7 |
| If the suicidal person has a weapon, the first aider needs to leave from the situation. | 20.5 |
| If the suicidal person has a weapon, the first aider needs to contact the police. | 58.8 |
| The first aider should avoid calling the police, unless absolutely necessary. | 67.6 |
| The first aider should only try to assist a suicidal person with whom they have a close relationship. | 26.4 |
| The first aider must not lie or use force to protect the life of the suicidal person. | 68.9 |
| *The first aider should contact the suicidal person’s spiritual or religious leader. | 58.6 |
|  |  |
| Section 5: Talking with a suicidal person **Accepted items:** |  |
| The first aider needs to allow the suicidal person to talk about their reasons for wanting to die. | 100.0 |
| The first aider should not argue with the person about their thoughts of suicide. | 94.1 |
| The first aider should encourage the suicidal person to do most of the talking. | 88.2 |
| The first aider should encourage the suicidal person to consider the consequences of suiciding, especially the effect it may have on the people s/he cares about. | 91.2 |
| The first aider should remind the suicidal person that these thoughts need not be acted on. | 94.1 |
| The first aider should suggest things to distract the suicidal person from these thoughts, especially things which are relatively easy to do and which will encourage a sense of control and achievement. | 82.7 |
| Suicidal thoughts are often a plea for help and a desperate attempt to escape from problems and distressing feelings. The first aider should therefore allow the suicidal person to talk about those feelings. | 100.0 |
| By discussing specific problems, the first aider can help the person work out ways of dealing with the difficulties that seem impossible to cope with. | 97.1 |
| The first aider needs to find out what has supported the suicidal person in the past, and whether these supports are still available. | 97.1 |
| The fact that the suicidal person is still alive, and talking to the first aider about their feelings, means that they are not quite sure about suicide. The first aider should point this out as a positive thing. | 89.3 |
| The first aider should ask the suicidal person what they think could help keep their mind away from negative thoughts. | 85.3 |
| The first aider should discuss the "good things" in a person's life, their hopes for the future, and other reasons to live. | 91.2 |
| The first aider should express empathy for the suicidal person. | 97.1 |
| The first aider should encourage the suicidal person to think about their personal strengths. | 91.2 |
| **Rejected items:** |  |
| To help the suicidal person change their mind, the first aider should tell them they will go to hell if they die by suicide. | 11.7 |
| To help the suicidal person change their mind, the first aider should tell them they ruin the lives of others if they die by suicide. | 20.6 |
| The first aider must not let the suicidal person draw them into discussion about whether suicide is right or wrong. | 68.9 |
| The first aider should reassure the suicidal person that thoughts of suicide are common, and that many people have them at some stage in their lives. | 55.9 |
| The first aider should accept the suicidal feelings for what they are and discuss suicide as a possibility rather than an unthinkable act. | 67.6 |
| It is important that the first aider dispute the idea that suicide is the best or the only solution to the person's problems. | 61.7 |
| The first aider should avoid giving advice. | 55.9 |
| The first aider should tell the suicidal person that the feelings they are experiencing are caused by a mental illness. | 14.7 |
| The first aider should tell the person to cheer up, and promise that everything will be okay. | 11.8 |
| In order to reduce suicide risk, it is important for the first aider to try to solve the suicidal person’s problems. | 14.7 |
| The first aider should help the suicidal person to put their problems into perspective by reminding them that other people have much worse problems and still choose to live. | 50.0 |
| The first aider should remind the suicidal person that they are loved and would be missed. | 67.6 |
| The first aider should remind the suicide person that suicide is a permanent solution to a temporary problem. | 38.2 |
| The first aider must avoid using glib expressions such as ‘suicide is a permanent solution to a temporary problem’. | 58.8 |
| The first aider should keep in mind that asking too many questions can provoke anxiety in the suicidal person. | 67.7 |
|  |  |
| Section 6: No-suicide contracts **Accepted items:** |  |
| The first aider should try to develop a contract with the suicidal person to ensure their safety. | 88.2 |
| Contracts should include an agreement that the suicidal person not attempts suicide. | 94.2 |
| Contracts should include safety contacts in case the suicidal person feels unable to continue with the agreement not to attempt suicide (such as a suicide helpline, professional helper or family member). | 100.0 |
| Contracts should include an agreement that the suicidal person not use any alcohol or other drugs. | 91.1 |
| Contracts should be for a length of time which will be easy for the suicidal person to cope with, so that they can feel able to fulfil the agreement and have a sense of achievement. | 91.1 |
| *Contracts should be phrased in a simple manner and easy to understand. | 93.1 |
| *The first aider should give a copy of the no-suicide contract to the suicidal person. | 89.7 |
| *Contracts should be developed together by the first aider and the suicidal person, so that both parties are agreed on the terms. | 86.2 |
| *The first aider should be aware that a no-suicide contract is not guarantee that the suicidal person will not kill themselves. | 93.1 |
| **Rejected items:** |  |
| The contract should be written down and signed by both the first aider and the suicidal person. | 78.6 |
| If the suicidal person doesn’t want to write the contract down, it is fine to make a verbal agreement. | 68.9 |
| Verbal agreements should be repeated out loud by the suicidal person back to the first aider to ensure that both parties are agreed on the terms. | 67.6 |
| Contracts should include an agreement that the suicidal person stops thinking about suicide. | 52.9 |
| Contracts should include an indication of when they will end, for example, the time when the suicidal person will next speak to the first aider. | 78.6 |
| Contracts should be for a length of time that will present a challenge for the suicidal person, so that they can see that they can manage alone. | 78.6 |
| A contract may be for as long as a week, if the suicidal person thinks they can manage that length of time. | 67.6 |
| A contract may be for as little time as one hour. | 29.4 |
| The first aider shouldn’t use a contract with a suicidal person they don’t know well. | 50.0 |
| The first aider shouldn’t use a contract with a suicidal person who is severely depressed. | 55.8 |
| The first aider shouldn’t use a contract with a suicidal person who is using drugs or alcohol. | 47.1 |
| The first aider shouldn’t use a contract with a suicidal person who is psychotic. | 64.7 |
| The first aider should only make a contract with someone they know well. | 47.0 |
| *Significant others (e.g. family members) should be involved in the development of the contract, to make it more appropriate and to increase compliance. | 69.0 |
| *A no-suicide contract may not work in Eastern cultures and has a debatable validity in such context. | 55.2 |
|  |  |
| Section 7: Ensuring safety **Accepted items:** |  |
| The first aider should remove the means of suicide available to the suicidal person. | 91.2 |
| If the first aider can’t get the suicidal person to agree to hand over the means of suicide (for example, pills, poison, gun, razors), emergency services must be contacted immediately. | 91.2 |
| If the suicidal person agrees to hand over the means of suicide, the first aider should take them and dispose of them right away (flush pills or poison down the toilet, hand gun to the police, throw away razors or knives). | 93.1 |
| The first aider must take steps to ensure the suicidal person will receive medical and/or psychological help once the immediate crisis has passed. | 100.0 |
| After the crisis has passed, the first aider should maintain contact (whether occasional or frequent, depending on the relationship) with the suicidal person. | 91.2 |
| **Rejected items:** |  |
| The first aider shouldn’t remove the means of suicide available to the suicidal person. | 8.8 |
| If the first aider can’t get the person to agree to hand over the means of suicide (for example, pills, poison, gun, razors) they should try to take these things secretly. | 52.9 |
| If the suicidal person agrees to hand over the means of suicide, on the condition that they can have them back if they want them, the first aider should argue the point with them for as long as it takes. | 29.4 |
| If the suicidal person agrees to hand over the means of suicide, on the condition that they can have them back on request, the first aider should agree to this. | 32.3 |
| If the intended means for suicide are of a less lethal type the first aider doesn’t need to worry about disabling the plan. | 20.5 |
| **Section 8: Confidentiality**  **Accepted items:** |  |
| A first aider must never agree to keep the risk of suicide a secret. | 96.6 |
| A first aider must never agree to keep the suicidal person’s suicidal plans a secret. | 82.3 |
| The suicidal person needs to be involved in decisions about who else knows about the suicidal crisis. | 93.1 |
| If the suicidal person refuses to involve someone else, the first aider must contact a professional. | 97.1 |
| *If the suicidal person is a minor, the first aider must make their guardians (i.e. the family or the social welfare) aware of the person’s intentions to kill themselves. | 100.0 |
| *If the first aider decides to involve a professional or someone else, they should inform the suicidal person of their decision and explain that this is necessary to ensure their safety. | 100.0 |
| *The first aider should tell the suicidal person’s immediate family about their intention to suicide. | 86.2 |
| *The first aider should ask for help from the person’s relatives, friends or housemates to ensure the person does not have access to weapons, poisons, or other means for suicide. | 100.0 |
| **Rejected items:** |  |
| If a suicidal person asks the first aider to promise they will keep the discussion about suicide a secret, the first aider should keep the secret. | 23.5 |
| If a suicidal person asks the first aider to promise they will keep the discussion about suicide a secret, the helper should agree, but tell someone else anyway. | 26.4 |
|  |  |
| **Section 9: Passing time during the crisis**  **Accepted items:** |  |
| During the suicidal crisis, the suicidal person and the first aider should be actively working on practical strategies to solve the life problems. | 87.9 |
| *During the suicidal crisis, the first aider should encourage the suicidal person to spend time with their significant others (e.g. family, friends or religious leader). | 93.1 |
| *It is preferable that the suicidal person chooses an activity which has been found in the past to help them to cope or that they enjoy. | 93.1 |
| **Rejected items:** |  |
| The first aider should encourage the suicidal person to take some sleeping pills, as they should be feeling better by the time they wake up. | 21.3 |
| The first aider should encourage the suicidal person to drink a few glasses of alcohol, to make the time pass more quickly. | 6.1 |
| The first aider should do something pleasant for the suicidal person. For example, cooking a favourite meal, renting a movie or listening to music with them. | 33.3 |
| The first aider should encourage the suicidal person to undertake some relaxing activities such as taking a hot bath, going for a long walk or reading something enjoyable. | 68.9 |
| The first aider should encourage the suicidal person to do something active like going for a swim or a jog. | 60.7 |
| If the suicidal person wants to be left alone, and can assure the first aider of their safety, the first aider should agree. | 33.4 |
| *During the suicidal crisis, the first aider should pray for or with the person. | 37.9 |
| *During the suicidal crisis, the first aider should encourage the suicidal person to spend time participating in their spiritual/religious and cultural practices (e.g., spend some time at church). | 41.4 |
| *The first aider and the suicidal person should find something to do together until the crisis has passed. | 62.1 |

1. The asterisk (*) indicates culturally specific items that were suggested by participants at Round 1. [↑](#footnote-ref-2)
