# Supplementary material for: Mental Health First Aid guidelines for helping a suicidal person: a Delphi consensus study in the Philippines
Source: Int J Ment Health Syst. 2010 Dec 20;4:32. doi: 10.1186/1752-4458-4-32 (PMC3017011; doi:10.1186/1752-4458-4-32)
Supplement: Additional file 2 — First aid guidelines for the Philippines. This file may be distributed freely, with the authorship and copyright details intact. Please do not alter the text or remove the authorship and copyright details. [file 1752-4458-4-32-S2.PDF]

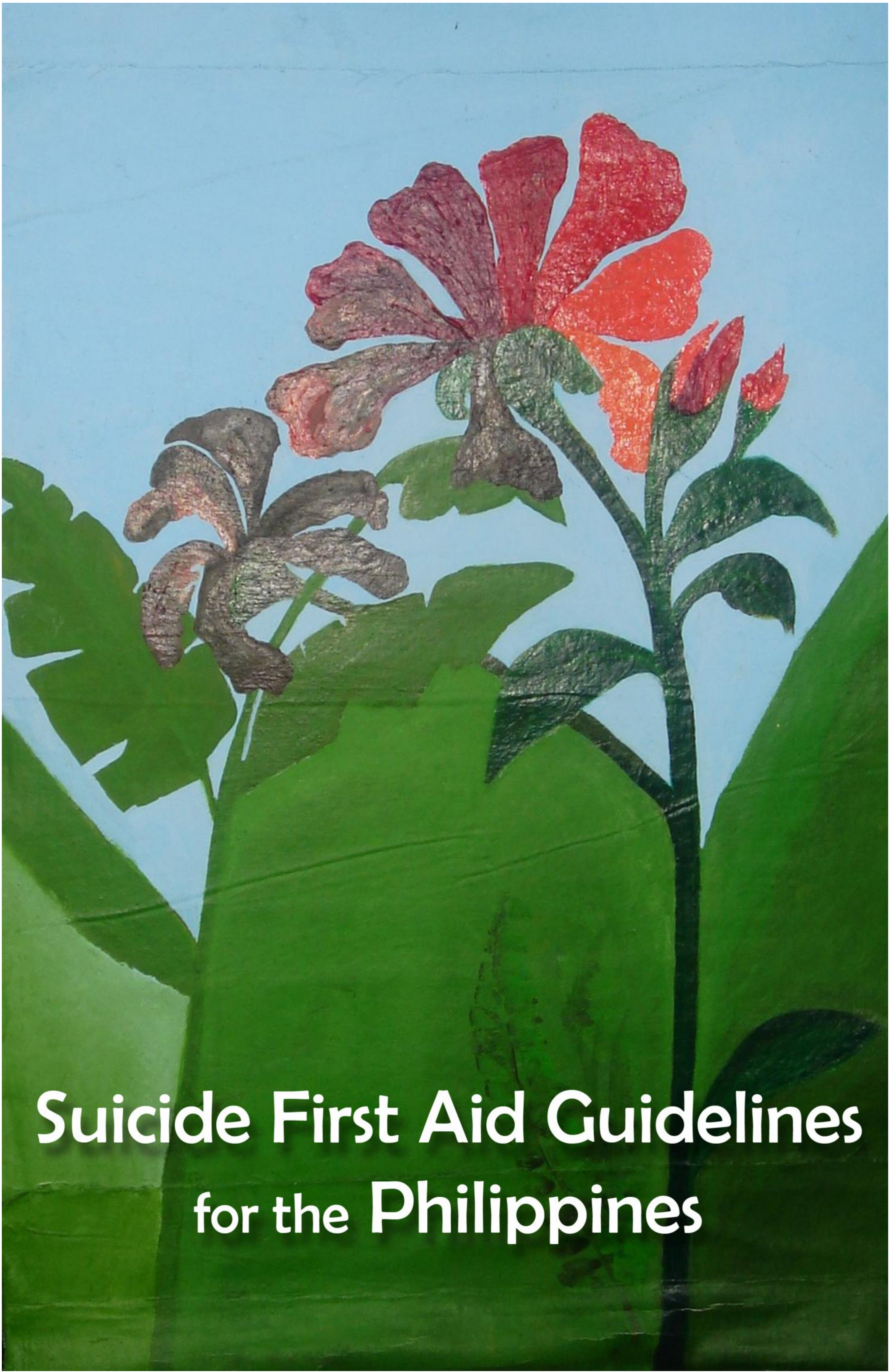

Suicide First Aid Guidelines for the Philippines

How can I tell if someone is feeling suicidal?

It is important that you are able to recognise the warning signs of suicide.

Signs a person may be suicidal

A dramatic change in mood, behaviour or appearance, for example:

- Expressing, in words or actions, hopelessness, worthlessness, guilt, shame, or having no reason to live or no purpose in life;
- Withdrawing from friends, family or society;
- Expressing, in words or actions, loss of interest in things that were previously of interest;
- Sudden or dramatic increase in depressed mood;
- Describing themselves as a burden to others or stating that others would be better off without them.

Someone who is suicidal may threaten to kill themselves, or say that they wish to die, verbally or in writing. This may be very direct, but is sometimes subtle. Watch for:

- Looking for a way to kill themselves (e.g. seeking access to pills or poisons, weapons or other means), including seeking information about possible suicide methods (e.g. would 100 mg of this kill me?);
- Unexpected jokes about death or suicide;
- Expressing, in words or actions, that they feel trapped, like there is no way out, or that suicide is the only solution to their problems;
- Expressing, in words or actions, the desire or hope that they will die (including praying that God may take their life).

People may also behave in ways that are life-threatening or dangerous, for example:

- Acting recklessly or engaging in risky activities, seemingly without thinking;
- Engaging in self-injurious behaviour such as cutting, poisoning or hitting their head against the wall;
- Stopping life-saving medical treatments/medications.

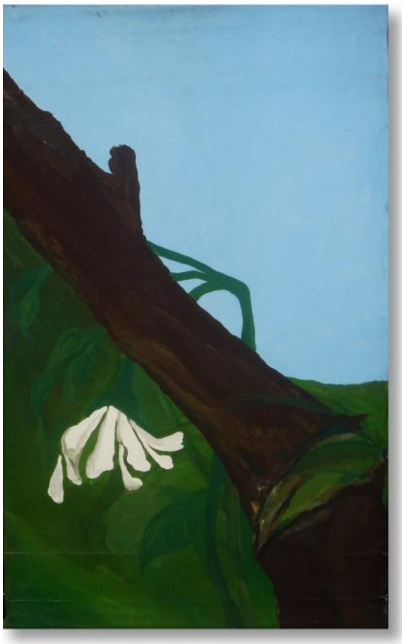

Someone who is suicidal may try to set their affairs in order, or make contact with peoplethey have not spoken to in some time, for example:

- Giving away valued possessions;
- Asking others to take on responsibility for the care of peo-ple or pets;
- Contacting people to say goodbye, make amends, or to ask for forgiveness.

People may show one or many of these signs whereas some may show signs not on this list (such as rage, anger, seeking revenge, anxiety, agitation, sleep disturbance, starting or increasing tobacco, alcohol or drug use; significant change in the level of religious interest or preoccupation with afterlife or any dramatic change in behaviour, mood, appearance).

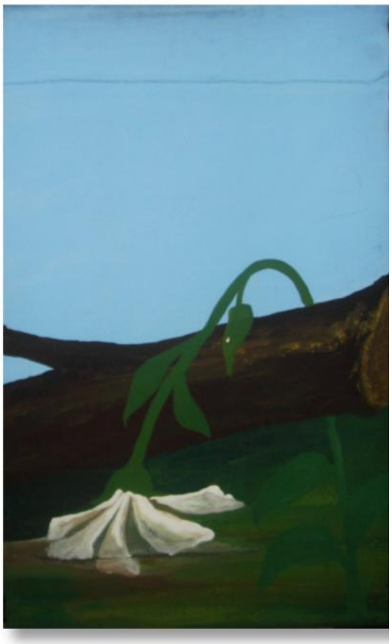

If you have seen some of these warning signs and you suspect that the person may be suicidal, you should ask them directly. For example, you might ask one of the following questions:

> Are you having suicidal thoughts?
>
> Are you thinking of killing yourself?

Don't avoid using the word "suicide." It is important to discuss the issue directly, without expressing fear or negative judgement. Doing so may help you to appear confident in the face of the suicide crisis, which may have a reassuring effect on the person you are helping.

However, if the person is using alcohol or other drugs, you may not be able to believe them if they say they are not suicidal. If you are still concerned, seek professional help.

Although some people think that talking about suicide can put the idea in the person's mind, this is not true. Another myth is that someone who talks about suicide is not really serious. Remember that talking about suicide may be a way for the person to indicate just how badly they are feeling.

How can you tell if the situation is serious?

First, you will need to know if a person has a clear intention to take his/her own life or whether they are more in an unsure state of suicidal thoughts like asking "what the point is?" or saying that " nothing can stop them". To do this you need to ask if they are planning to complete suicide. The three questions that you need to ask are:

> 1. Have you decided how you would kill yourself?
> 2. Have you decided when you would do it?
> 3. Have you taken any steps to secure the things you would need to carry out your plan?

A higher level of planning indicates a more serious risk. However, you must remember that the absence of a plan is not enough to ensure the person's safety. **All thoughts of suicide must be taken seriously.**

You need to know about the following additional risk factors:

- Has the person been using alcohol or other drugs? The use of alcohol or other drugs can make it more likely that a person will act on impulse.
- Has the person made a suicide attempt in the past? A previous suicide attempt makes it more likely that a person will attempt suicide again or kill themselves. You should ask the person directly if they have made a suicide attempt in the past, or ask their significant others (e.g. family members, close friend or religious leader).
- Does the person know anyone who has died by suicide? Having a family member or close friend die by suicide increases the risk.
- Once you have established that the risk of suicide is present, you need to take action to keep the person safe.

How can I keep the person safe?

Never leave someone who is feeling suicidal on their own. You do not have to be with the person all the time, but you should check on them regularly. Try to engage other people from the person's social network in preventing suicide. Ask the person if there is someone they can turn to when they need help or support. You should also ask the person if they would like you to contact someone for them, such as a friend, family member, trusted religious leader or spiritual adviser.

Try to remove the means of suicide available to the person if it is safe to do so. Ask the person to give you the things they intend to use to kill themselves. If they agree, you should dispose of them right away (flush pills or poison down the toilet, give gun to the police, safely dispose of razors or knives). If they do not agree, call emergency services such as the police. If there is any risk to your safety (for example, if the person has a gun or other weapon or is agitated), do not attempt to remove the means of suicide. In this case, call the police right away.

Tell the person's immediate family about their intention to suicide, particularly if the person is a minor (i.e. under the legal age for adulthood). Ask for help from their relatives, friends or housemates to ensure the person does not have access to weapons, poisons, or other means for suicide. It is better to work collaboratively with the person and others to ensure their safety, rather than acting alone to prevent suicide at any cost.

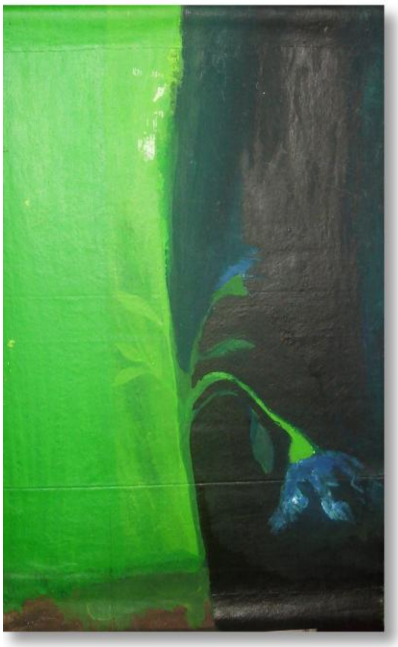

What about professional help?

Make sure that you have the phone numbers of suicide hotlines, emergency services and mental health professionals on hand in case of emergency. You must call or take the person to a doctor, psychiatrist or other mental health professional right away. Call a mental health emergency team, or whoever is responsible for responding to psychiatric emergencies. In particular, if the suicidal person is suffering from psychosis, or using alcohol or other drugs, emergency assistance should be sought immediately. If emergency help is likely to take a long time to arrive, take the suicidal person to the nearest safe place (e.g. church, hospital or police station).

The person needs to be involved in decisions about who else knows about their thoughts of suicide. However, if they refuse to involve someone else, you should still contact a professional. You should explain that this is necessary to ensure the person’s safety. You must never agree to keep the person's suicidal thoughts or plans a secret.

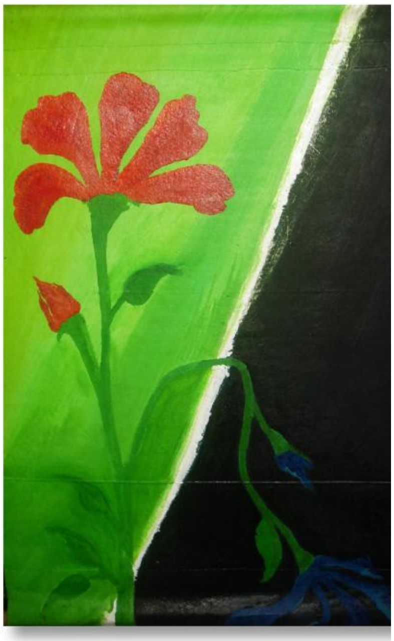

How should I talk with someone who is suicidal?

Express empathy for the person and tell them that you care and want to help. You should remain calm and in control, and try to appear confident, \as this can be reassuring for the person. Encourage the person to do most of the talking and listen to them without expressing judgment. You should not argue with the person about their thoughts of suicide.

The threat of suicide may indicate that a person is trying to communicate how badly he or she feels, and is often a plea for help and a desperate attempt to escape from problems and distressing feelings. You should therefore allow the person to tell about those feelings and their reasons for wanting to die.

Help the person to understand that they have control over their suicidal thoughts, and that these thoughts do not need to be acted on. Point out to the person the fact that s/he is still alive and talking to you about their feelings means that they are not quite sure about suicide and that this is a positive thing.

Thoughts of suicide are sometimes caused by a mental disorder, so you should find out if the person has such a disorder. Avoid asking if they have a ‘mental illness’, instead ask if they are receiving help for any emotional or mental health problems. Clearly state that thoughts of suicide may be caused by a treatable disorder, as this may instill a sense of hope for the person.

By discussing specific problems, you can help the person work out practical strategies for effectively dealing with difficulties and life problems that seem impossible to cope with. Find out what has supported the person in the past, and whether these supports are still available.

Consider and use the person’s belief systems and values, including their spiritual and religious beliefs, to encourage them to change their mind about suicide.

Ask the person what they think could help keep their mind away from negative thoughts. Find out whether there is anything important in the person’s life which may reduce the immediate risk of suicide (e.g. attachments to children). Encourage the person to consider the consequences of suicide, especially the effect it may have on the people s/he cares about. However, do not make the person feel guilty or ashamed. Instead, focus on the importance of the person in the lives of the people they care about, the "good things" in the person's life, their hopes for the future, and other reasons to live. Encourage the person to think about their personal strengths.

No-suicide contracts

Try to develop a contract with the person to ensure their safety. A contract is an agreement not to act on thoughts of suicide. Be aware that a no-suicide contract is not a guarantee that the person will not kill themselves, and it is not a legal document. However, it can be a useful tool. The contract should be phrased in a way which is simple to understand.

The contract should be for a length of time which is easy for the person to cope with (even just a few hours), so that they can feel able to fulfil the agreement and have a sense of achievement. It should include safety contacts (such as a suicide helpline, professional helper or family member) in case the person feels unable to continue with the agreement not to attempt suicide. It should also include an agreement not to use any alcohol or other drugs.

If it is written down, you should give a copy of the no-suicide contract to the person so that they know exactly what is being agreed to.

Passing time during the crisis

Suicidal crises can last a few hours or a few days. While the person is suicidal, whether you remain with them all the time (as a family member or friend) or only for a short time, there is time to pass. You will need to find a way to pass this time. Suggest things to distract the person from their suicidal thoughts, especially things which are relatively easy to do and which will encourage a sense of control and achievement. It is preferable that the person chooses an activity which has been found in the past to help them to cope or that they enjoy. Encourage the person to spend time with their significant others (e.g. family, friends or religious leader).

After the crisis has passed, maintain contact (whether occasional or frequent, depending on the relationship) with the person and take steps to ensure they are receiving professional help.

A final note:

Remember that despite your best efforts, some people will still die by suicide. However, always do your best for the person you are helping.

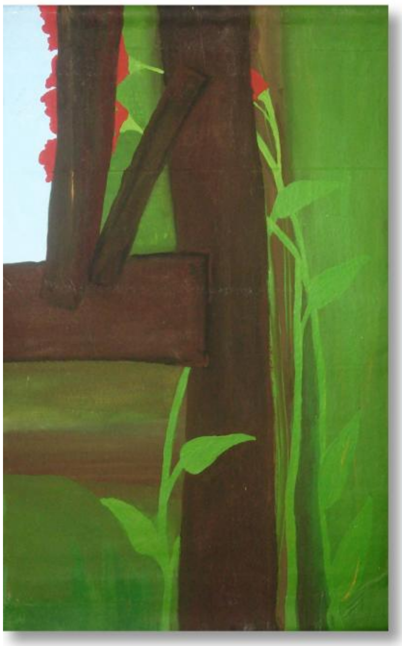

An important note:

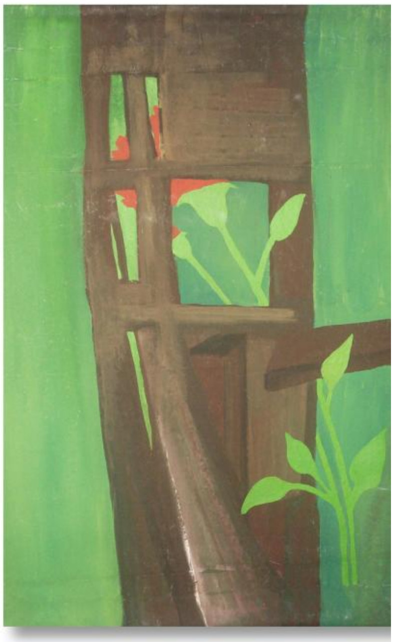

Self-injury can indicate a number of different things. Someone who is hurting themselves may be at risk of suicide. Others engage in a pattern of self-injury over weeks, months or years and are not necessarily suicidal.

It is not easy to tell the difference between self-injury and a suicide attempt. Some people argue that anyone who injures themselves must be suicidal even if they are not conscious of it. Others say that it is the person's intentions which count. The only way to know is to ask the person directly if they are suicidal. These guidelines can assist you only if the person you are helping is suicidal. If the person you are assisting is injuring themselves,but is not suicidal, please refer to the guidelines entitled MHFA Guidelines for non-suicidal self-injury.

Purpose of these Guidelines

These guidelines are designed to help members of the public to provide first aid to someone who is at risk of suicide. The role of the first aider is to assist the person until appropriate professional help is received or the crisis resolves.

Development of these Guidelines

The following guidelines are created based on the expert opinions of a panel of mental health professionals from the Philippines on how to help someone who may attempt to commit suicide. Details of the methodology will be published in the International Journal of Mental Health Systems. Please visit our website at www.ijmhs.com to download a free copy or you may contact us to request for a copy.

How to use these Guidelines

These guidelines are a general set of recommendations about how you can help someone who may be at risk of suicide. Each individual is unique and it is important to tailor your support to that person's needs. Also, the guidelines are designed to be suitable for providing first aid in the Philippines. They may not be suitable for other cultural groups or for countries with different health systems.

Although these guidelines are copyright, they can be freely reproduced for non-profit purposes provided the source is acknowledged. Please cite these guidelines as follows:

Colucci, E., Kelly, C., Minas, H.K. and Jorm, A.F., Suicide First Aid Guidelines for the Philippines. Melbourne: Centre for International Mental Health & ORYGEN Youth Health Research Centre, The University of Melbourne; 2009.

Enquiries and expressions of interest to organize Suicide First Aid training should be sent to:

Dr Erminia Colucci, CIMH, School of Population Health, The University of Melbourne,
Level 5, 207Bouverie St 3053 Carlton Melbourne VIC Australia
Email: ecolucci@unimelb.edu.au or fera_76@hotmail.com

All MHFA guidelines can be downloaded from www.mhfa.com.au/Guidelines.shtml. Please check this website for updates on this guidelines.

This research was supported by a grant from the American Foundation for Suicide Prevention and CIMH funds.

Before distributing these guidelines, please ADD HERE help-services' contact numbers (e.g. help-lines, mental health emergency team, or a mental health professionals association). FREE or REFUNDABLE help-services are preferred. Thanks!

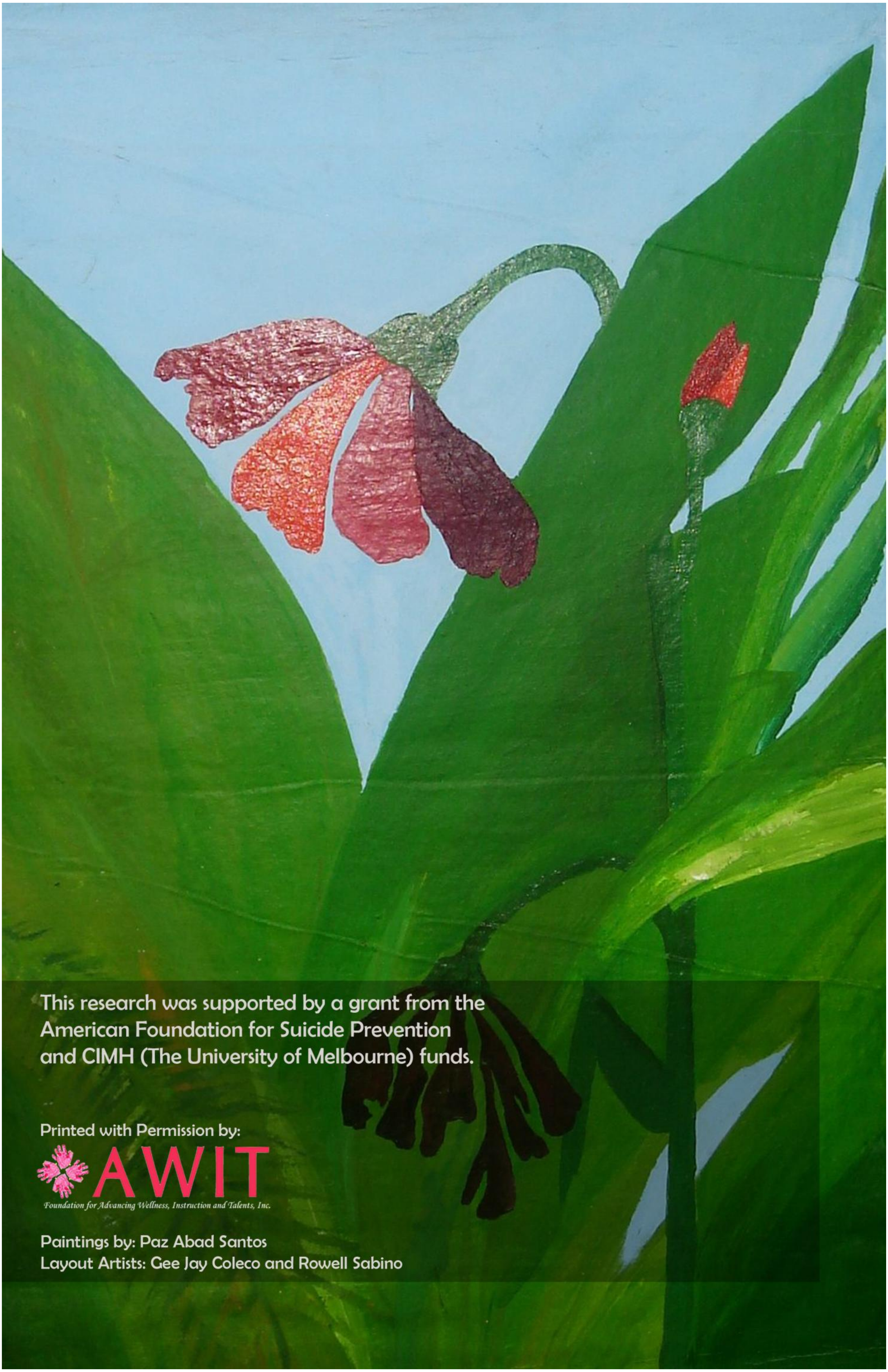

This research was supported by a grant from the American Foundation for Suicide Prevention and CIMH (The University of Melbourne) funds.

Printed with Permission by:

AWIT
Foundation for Advancing Wellness, Instruction and Talents, Inc.

Paintings by: Paz Abad Santos
Layout Artists: Gee Jay Coleco and Rowell Sabino
